# Bone-Marrow-Derived Mesenchymal Stem Cells Attenuate Behavioral and Cognitive Dysfunction after Subarachnoid Hemorrhage via HMGB1–RAGE Axis Mediation

**DOI:** 10.3390/life13040881

**Published:** 2023-03-26

**Authors:** Harry Jung, Dong Hyuk Youn, Jeong Jin Park, Jin Pyeong Jeon

**Affiliations:** 1 Institute of New Frontier Research Team, Hallym University College of Medicine, Chuncheon 24252, Republic of Korea; 2 Department of Neurology, Konkuk University Medical Center, Seoul 05030, Republic of Korea; 3 Department of Neurosurgery, Kangwon National University College of Medicine, Chuncheon 24341, Republic of Korea; 4 Department of Neurosurgery, Hallym University College of Medicine, Chuncheon 24253, Republic of Korea

**Keywords:** subarachnoid hemorrhage, bone-marrow mesenchymal stem cells, cognitive impairment, high-mobility group box 1

## Abstract

We evaluated the therapeutic effects of bone-marrow-derived mesenchymal stem cells (BMSCs) on behavioral and cognitive function in a mouse model of mild subarachnoid hemorrhage (SAH) and explored the underlying mechanisms in conjunction with the HMGB1–RAGE axis. The SAH models were generated in a total of 126 male C57BL/6J mice via endovascular perforation and evaluated 24 h and 72 h after the intravenous administration of BMSCs (3 × 10^5^ cells). The BMSCs were administered once, at 3 h, or twice, at 3 h and 48 h after the model induction. The therapeutic effects of the BMSCs were compared to those of the saline administration. Compared to saline-treated SAH-model mice, at 3 h, the mice with mild SAH treated with the BMSCs showed significant improvements in their neurological scores and cerebral edema. The administration of the BMSCs decreased the mRNA expression of HMGB1, RAGE, TLR4, and MyD88, as well as the protein expression of HMGB1 and phosphorylated NF-kB p65. Furthermore, the numbers of slips per walking time, impairments in short-term memory, and the recognition of novel objects were improved. There was some improvement in inflammatory-marker levels and cognitive function according to the BMSCs’ administration times, but no large differences were seen. The administration of BMSCs improved behavioral and cognitive dysfunction by ameliorating HMGB1–RAGE axis-mediated neuroinflammation after SAH.

## 1. Introduction

Subarachnoid hemorrhage (SAH) is a neurological disease with a high rate of in-hospital mortality, which ranges from 20 to 30%. The pooled incidence of SAH was reported to be 21.4 per 100,000 person-years [1,2]. The main focus of research on SAH is on improving patient survival by effectively preventing rebleeding (clipping vs. coiling) and minimizing neurological complications, such as delayed cerebral ischemia (DCI) and hydrocephalus. However, although favorable neurological outcomes are achieved by the use of appropriate treatments, a significant number of SAH survivors complain of behavioral deficits or changes such as motor weakness, sensory changes, and balance problems, and exhibit cognitive dysfunction in various domains of visual and verbal memory, as well as a decline in executive functions [3]. Hunt–Hess grades greater than 2, a thick SAH, and DCI are well-known risk factors for behavioral and cognitive dysfunction [4,5]. Subarachnoid hemorrhage can result in behavioral and cognitive dysfunction by altering functional connectivity [6]. Despite good neurological outcomes, persistent behavioral and cognitive dysfunction impairs quality of life. Nevertheless, the research on therapeutics for SAH patients is limited compared to the research on therapies for those with neurodegenerative diseases or ischemic stroke.

Stem-cell treatments have been investigated for use in treating some brain diseases, especially degenerative brain diseases, in anticipation of replacing injured neuronal cells and reconstructing damaged neural circuits using stem cells [7,8,9]. Among various types of stem cell, mesenchymal stem cells (MSCs) are advantageous in terms of immune tolerance because they express fewer major histocompatibility complex class I molecules and are easily obtained and handled [10]. Previous studies on MSC treatments for SAH have focused on ameliorating neuroinflammation in the early phase after the ictus of SAH [11,12]. Khalili et al. [11] first reported that MSC treatment 24 h after SAH significantly improved functional recovery and reduced apoptosis. Liu et al. [12] reported that bone-marrow-derived stem-cell (BMSC) treatment 1 h after SAH contributed to decreased brain water content and blood–brain barrier (BBB) permeability by inhibiting Notch1-dependent neuroinflammation. However, few studies have examined whether MSC treatment is linked to the attenuation of behavioral and cognitive dysfunction. Neuroinflammation itself is widely recognized as a potential mediator of behavioral and cognitive dysfunction [13]. In particular, high-mobility-group box protein 1 (HMGB1) has received attention in the study of various disease conditions, since it is released by glial cells and neurons after brain damage [14]. The HMGB1 continuously binds to toll-like receptors (TLRs) and the receptor for advanced glycation end products (RAGE), activating inflammatory responses [14]. The anti-HMGB1 antibody improved behavioral performance and reduced oxidative stress in injured brains after hemorrhage [15]. The suppression of HMGB1, TLR, and RAGE was shown to halt the progression of amyloid-beta (Aß) loading in Alzheimer’s disease (AD) and diabetes-related dementia [16,17]. Thus, we investigated the therapeutic effects of BMSCs on behavioral and cognitive dysfunction after SAH and explored the underlying mechanisms in conjunction with HMGB1-mediated neuroinflammation in an experimental mouse model of mild SAH.

## 2. Materials and Methods

### 2.1. Experimental SAH Model

The C57BL/6J male mice, 8–10 weeks of age and weighing 20–25 g, were obtained from the Laboratory Animal Resources Center of Hallym University. The animals were provided with regular food and water ad libitum under a 12-h dark/light cycle at 24 °C and 55 ± 10% humidity. The SAH model was generated using the endovascular perforation technique, as described previously [4,18]. The mice were anesthetized with 2.5% isoflurane in oxygen. Next, a vertical incision was made on the midline of the neck, and the external and common carotid arteries were exposed. Subsequently, sharpened 5-0 Prolene suture material was inserted in the external carotid artery and advanced, ultimately perforating the distal internal carotid artery (ICA), which is divided into the anterior and middle cerebral arteries. Mice in the sham-operated group underwent the same procedure, except for arterial perforation [19]. In this study, we used only mild-SAH-model mice evaluated at 24 h based on modified Garcia neurological scale scores of 16 and 17 [19,20]. At the end of the study, mice were hyperanesthetized with 3.5% isoflurane in oxygen for 5 min. After confirming that each mouse’s heart had stopped, the brain was isolated. For histology analysis, mice were anesthetized with 2.5% isoflurane in oxygen followed by cardiac perfusion before their brains were collected. After separating the mouse brains, brain-tissue samples, from which the olfactory bulb and cerebellum were removed using a microblade, were used for analysis. Next, the isolated tissues were homogenized using Omni Bead mill homogenizer (Omni International, Kennesaw, GA, USA). All animal experiments were approved by the Institutional Animal Care and Use Committee (IACUC) of Hallym University (no: HallymR1 2021-50).

### 2.2. BMSC Culture

The BMSCs isolated from normal human bone marrow without disease were purchased from ATCC (PCS-500-012, Manassas, VA, USA). We confirmed BMSCs with pluripotent marker expression of SOX2, OCT4, and Nanog for at least 5 passages. We cultured BMSCs by referring to previous papers [21,22]. Cells were plated at a density of 1 × 10^6^ cells in a 100-mm culture dish in high-glucose Dulbecco’s modified Eagle medium (DMEM), 10% fetal bovine serum (FBS), and 100 U/mL penicillin/streptomycin (Gibco, Grand Island, NY, USA). After 48 h, nonadherent cells contained in the medium were removed, and fresh culture medium was added to the adherent mononuclear cells, which represented BMSCs. All BMSCs used in this experiment were from passage 5. The BMSCs (3 × 10^5^ cells) were administrated intravenously to the mice.

### 2.3. BMSC Administration

A total of 126 male C57BL/6J mice were randomly divided into 6 groups (Supplemental Figure S1). After SAH induction, BMSCs were administered to the mice once, at 3 h, or twice, at 3 h and 48 h, and the treatment effects were evaluated at 24 h and 72 h, respectively. In detail, the analysis groups were: (1) normal controls (Normal); (2) SAH mice treated with saline at 3 h and evaluated at 24 h (24 h-S-3 h); (3) SAH mice treated with BMSCs at 3 h and evaluated at 24 h (24 h-B-3 h); (4) SAH mice treated with saline at 3 h and evaluated at 72 h (72 h-S-3 h); (5) SAH mice treated with BMSCs at 3 h and evaluated at 72 h (72 h-B-3 h); (6) SAH mice administered saline twice, at 3 h and 48 h, and evaluated at 72 h (72 h-S-3 h/48 h); and (7) SAH mice treated with administered BMSCs twice, at 3 h and 48 h, and evaluated at 72 h (72 h-B-3 h/48 h). The results were compared to those of mice administered saline.

### 2.4. RNA Isolation and Quantitative RT-PCR

Total RNA in the brain tissues was extracted using easy-BLUE Total RNA Extraction Kit and reverse-transcribed into cDNA using Maxime RT PreMix Kits (iNtRON Biotechology, Inc., Santa Cruz, MA, USA). The mRNA purity was measured using an Eppendorf BioSpectrometer Basic (Eppendorf, Hamburg, Germany). The A260/A280 ratio was used in this study to indicate the mRNA purity, which ranged from 1.9 to 2.0. Quantitative real-time polymerase chain reaction (qRT-PCR) analysis was performed in triplicate for each sample using SYBR Green PCR Kits (Applied Biosystems, Foster City, CA, USA) for 60 cycles with a 3-step program of 15 s of denaturation at 94 °C, 30 s of annealing at 55 °C, and 30 s of extension at 70 °C. Amplification specificity was assessed by melting-curve analysis. The sequences of the qRT-PCR primers are presented in Supplemental Table S1. Glyeraldehyde-3-phosphate dehydrogenase (GAPDH) was used as the endogenous control. The qRT-PCR results were analyzed using the 2^−ΔΔct^ method.

### 2.5. Western Blots

After lysing brain tissues in radio-immunoprecipitation assay buffer supplemented with a proteinase-inhibitor cocktail, the protein concentrations were measured using the Pierce BCA Protein Assay Kit (Thermo Fisher Scientific Inc., Waltham, MA, USA). Proteins (15 ug) were separated by 10–15% sodium dodecyl sulfate–polyacrylamide-gel electrophoresis (SDS PAGE) and transferred onto polyvinylidene-fluoride membranes. The membranes were blocked in 1% bovine serum albumin (BSA) for 1 h at room temperature and incubated with primary antibodies overnight at 4 °C. The following primary antibodies were used: interleukin (IL)-6 (1:500, Santa Cruz Biotechnology, Dallas, TX, USA), tumor necrosis factor (TNF)-α (1:500, Santa Cruz Biotechnology), COX-2 (1:1000, Abcam, Waltham, MA, USA), HMGB1 (1:1000, Cell Signaling Technology, Danvers, MA, USA), nuclear factor (NF)-kB p65 (1:1000, Cell Signaling Technology), and phosphor-NF-kB p65 (1:1000, Cell Signaling Technology). The membranes were washed three times for 15 min in tris-buffered saline with 0.1% Tween 20 and then incubated with HRP-linked secondary antibody. The bots were exposed to X-ray film for 1–5 min and analyzed by ImageJ software (Image J 1.49v, National Institutes of Health). Rationales for investigating various markers used in this study are presented in the Supplemental Method Section.

### 2.6. Brain Water Content

The wet weights of the mouse brains were obtained and the brains were dried in the oven at 100 °C for 3 h to obtain the dry weights, as in a previous report [23]. Cerebral edema was measured by the following formula: brain water content percentage = [(wet weight − dry weight)/wet weight] × 100% [23].

### 2.7. Behavioral and Cognitive Dysfunction

Behavioral assessments were conducted using modified Garcia neurological scale scores and the beam-walking test, according to previous studies [4,24]. The beams were located horizontally and 50 cm above the table. Briefly, in the beam-walking test, mice were trained to cross an illuminated alley (10 cm wide and 33 cm long) and move straight to the opposite side, located at the end of the alley. Their hindlimbs were then coated with non-toxic ink, and the mice were allowed to walk through a small tunnel on a sheet of white paper. This results of 1–2 trials were recorded until 10 clearly visible footprints per animal were obtained. Mice were trained to traverse a beam 2 cm in diameter and then a beam 1 cm in diameter. The latency time to traverse each beam and latency time to fall were recorded. Cognitive dysfunction was assessed using Y-maze and novel object recognition (NOR) tests. The Y-maze test was used to assess short-term spatial memory. Each mouse was placed in a white Y-maze with three arms, which were designated A, B, and C. Each arm was 40 cm long, 12 cm high, and 10 cm wide. Each mouse was placed at the end of the starting arm and allowed to move freely through the maze for 5 min. Alternation behavior was defined as consecutive entries into three arms. The major outcome was spontaneous alternation (alternation index = alternation/maximum alternation × 100). The NOR test was performed according to our previous report (Supplemental Data) [23]. The mice were habituated for 10 min for two days in an open square field (65 × 45 × 30 cm) and then trained with two identical objects (A1 and A2) in opposite quadrants in the arena. After 24 h, the mice were exposed to a familiar object (A1) and a novel object (B1) for 10 min. Twenty-four h and seventy-tow h after the SAH, mouse activity was evaluated by changing the distance between A1 and the novel object (C1) in the 10-min open-field test. Object preference was measured once, at s distance between the mouse’s nose and the object of less than 2 cm. All mouse-behavior tests were recorded by a video tracking system and described using heatmap image tracking (NoldusEthoVision XT, Leesburg, VA, USA) [23].

### 2.8. Statistical Analysis

All data are presented as the means with standard errors of the mean (SEM). One-way analysis of variance (ANOVA) with post hoc Bonferroni correction was conducted for all possible pairwise comparisons [25]. Any *p*-values of less than 0.05, 0.01, and 0.001 are represented by *, **, and *** in the figures, respectively [26]. All statistical analyses were conducted using GraphPad Prism software (v.8.0; GraphPad Software Inc., San Diego, CA, USA).

## 3. Results

### 3.1. Effect of BMSC on the In Vivo SAH Model

**Figure 1:**
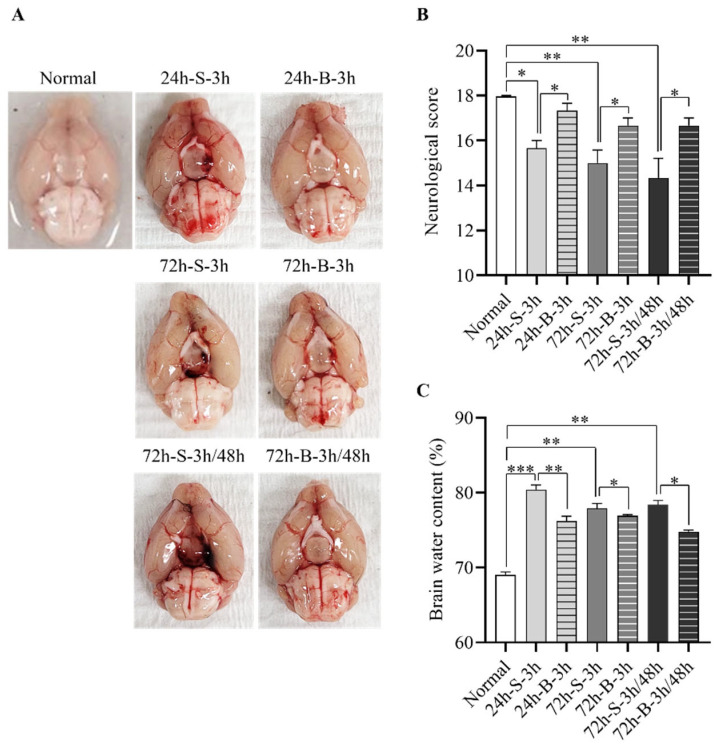
Therapeutic effects of BMSC on the in vivo SAH model. (**A**) Representative brain images of the normal control and in vivo SAH model mice treated with saline or BMSCs observed at 24 h or 72 h after model induction. (**B**,**C**) Comparison of neurological scores (n = 6) and brain water content (n = 6) according to the treatment method. * *p* < 0.05, ** *p* < 0.01, and *** *p* < 0.001. The data represent the mean ± standard error of the mean (SEM).

We explored the therapeutic effects of the BMSCs on neurological deficits and cerebral edema after the SAH induction in the mice (Figure 1 and Figure S1). Compared to the control group, the mice with mild SAH administered saline at 3 h exhibited definite blood clots in the basal cistern and decreased neurological scores with increased cerebral edema at 24 h and 72 h. In particular, although the amount of clotted blood decreased visually at 72 h, the neurologic scores and edema continued to deteriorate. The mice with mild SAH and BMSC administration at 3 h showed significantly improved neurological scores with decreased cerebral edema compared to the mice with mild SAH and saline administration (17.3 ± 0.6 in the BMSC treatment group vs. 15.7 ± 0.6 in the saline treatment group; *p* < 0.005). When measured at 72 h, the neurologic scores and cerebral edema showed no significant improvements compared to the measurements at 24 h. To examine the differences in the therapeutic effects at 72 h according to the number of administrations, the BMSCs were administered twice, 3 h and 48 h after the model induction. Contrary to our expectations, similar effects on the neurological scores and brain water content were seen in the mice with mild SAH treated with BMSCs twice compared to a single administration of BMSCs.

### 3.2. Histological Examination, Neuronal Apoptosis, and Inflammation

**Figure 2:**
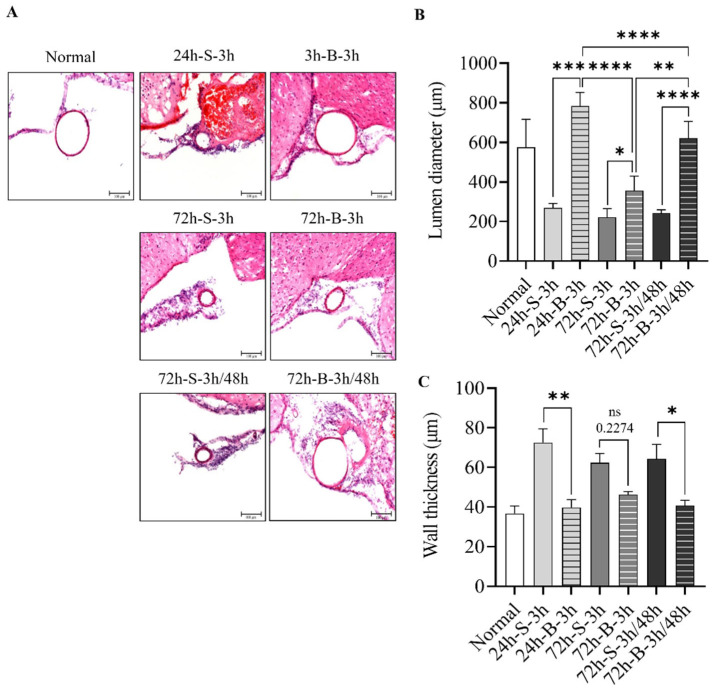
Change in histological examination, lumen size, and wall thickness for BMSC in the in vivo SAH model. (**A**–**C**) Representative hematoxylin and eosin-stained coronal sectioned images of the distal internal carotid artery according to treatment method, such as saline or BMSC, observed at 24 h or 72 h after SAH induction (n = 6). Error bars, mean SEM, ns: not significant, * *p* < 0.05, ** *p* < 0.01, *** *p* < 0.005, and **** *p* < 0.001.

The H&E staining performed 24 h after SAH induction showed that the mice with mild SAH and saline administration had decreased luminal areas in the distal ICA, with increased wall thickness compared to those administered the BMSCs (Figure 2A). More pronounced thick blood in the subarachnoid space and thick subarachnoid layers over the convexities were observed at 24 h after the SAH in the saline-treated group compared to the BMSC-treated group. The H & E staining at 72 h showed thin layers on the convexities and decreased numbers of invading cells under the pia mater after the administration of the BMSCs. The lumen diameter of the distal ICA was 576.4 ± 75.1 μm for the normal mice. It was decreased to 269.8 ± 10.9 μm at 24 h after the SAH induction. The wall thickness of the distal ICA was 36.8 ± 3.8 μm for the normal mice. It increased to 72.5 ± 7.0 μm at 24 h after the SAH induction. Anatomically, the lumen area and the thickness of distal ICA were similar to those of the normal-mice group after the administration of the BMSCs at 3 h and 48 h after the SAH induction. At 72 h after the SAH induction, the wall thickness was 46.3 ± 1.6 μm for the single-BMSC-administration group and 62.3 ± 4.8 μm for the saline-administration group (Figure 2B,C).

**Figure 3:**
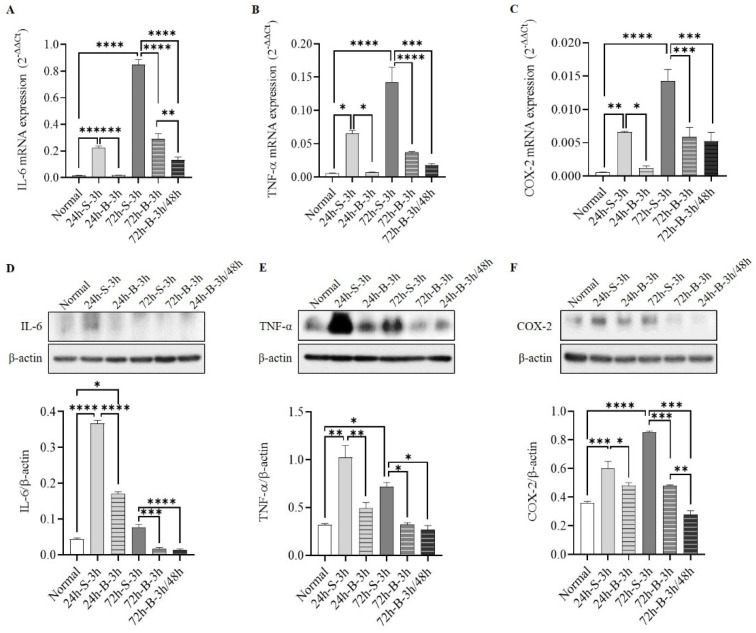
Comparisons of mRNAs (**A**–**C**) and proteins (**D**–**F**) of IL-6, TNF-α, and COX-2 in in vivo SAH model mice according to the treatment method (n = 6). Error bars, mean SEM, * *p* < 0.05, ** *p* < 0.01, *** *p* < 0.005, and **** *p* < 0.001.

The mRNA- and protein-expression levels of IL-6, TNF-α, and COX-2 were significantly decreased after the BMSC administration at both 24 h and 72 h after the SAH induction compared to the control group (Figure 3A–F). In particular, the IL-6 and COX-2 proteins decreased more after two BMSC administrations than after one.

### 3.3. Changes in HMGB1–RAGE Axis Inflammation

**Figure 4:**
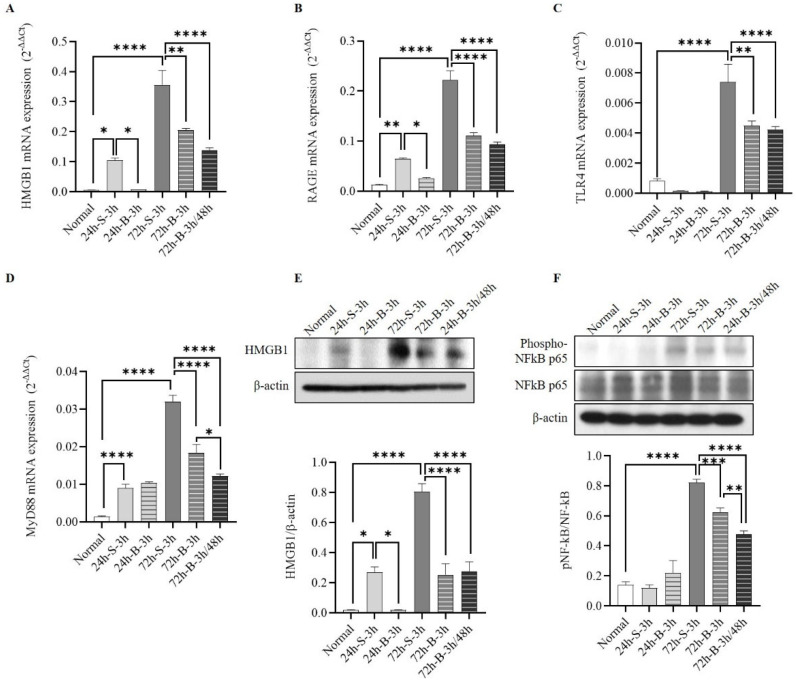
Changes in HMGB1–RAGE axis inflammation. (**A**–**D**) The mRNA expression of HMGB1–RAGE inflammation by HMGB1, RAGE, TLR4, and MyD88 using qRT-PCR in in vivo SAH-model mice based on treatment. (**E**,**F**) Comparison of HMGB1, total NF-kB p65, and phospho-NF-kB p65 according to the treatment method (n = 6). Error bars, mean SEM, * *p* < 0.05, ** *p* < 0.01, *** *p* < 0.005, and **** *p* < 0.001.

To determine inflammatory changes in the HMGB1–RAGE axis after the BMSC administration, we measured the mRNA-expression levels of HMGB1, RAGE, TLR4, and MyD88 using qRT-PCR, and the protein-expression levels of HMGB1, total NF-kB p65, and phospho-NF-kB p65 by Western blotting. The BMSC administration decreased the mRNA expression of HMGB1, RAGE, TLR4, and MyD88 after the SAH induction (Figure 4A–D). Reductions in TLR4 and MyD88 mRNA expression were seen at 72 h. The protein expressions of HMGB1 and phosphorylated NF-kB p65 decreased after the BMSC administration (Figure 4E,F). In particular, NF-kB p65 decreased more when the BMSCs were administered twice compared to once.

### 3.4. Behavior and Cognition

**Figure 5:**
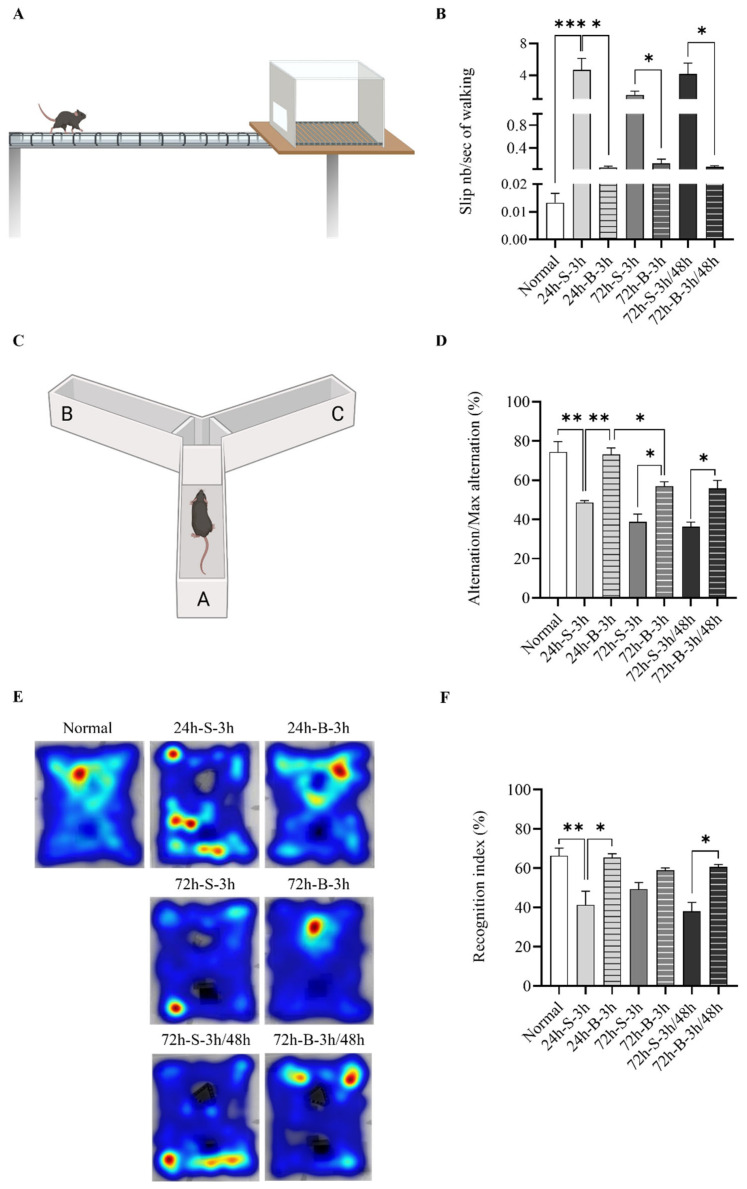
Effect of BMSC on behavior and cognitive dysfunction in in vivo SAH-model mice analyzed by beam-walking (**A**,**B**), the Y-maze (**C**,**D**), and NOR tests (**E**,**F**) (n = 9). Error bars, mean SEM, * *p* < 0.05, ** *p* < 0.01, and *** *p* < 0.005. The data represent the mean ± SEM.

The BMSC administration improved the motor activity and balance (Figure 5 and Figure S2). The slip number/sec for walking in the normal mice was 0.013 ± 0.003 n/sec. It increased to 4.667 ± 1.258 n/sec at 24 h after the SAH induction in the saline-administered mice (Figure 5A,B). The alteration/maximum alteration rate in the normal mice was 74.4 ± 4.6%. It decreased to 48.6 ± 0.9% at 24 h after the SAH induction in the saline-administered mice. In particular, more improvements were seen after administering the BMSCs twice, at 3 h and 48 h, compared to once (Figure 5C,D). On the NOR test, the percentage of identical object (A1 and A2) recognition after the training phase and the percentage of familiar object (A1) and novel object (B1) recognition did not significantly differ between the groups (Supplemental Figure S2). After the SAH induction, the recognition index of a novel object (C1) by the SAH-model mice treated with the BMSCs at 3 h was significantly higher than in those treated with the saline at 24 h. The novel recognition rate was higher when the BMSCs were administered twice, at 3 h and 48 h, compared to the saline-administered group, but there was no significant difference compared to the group administered BMSCs once, at 3 h (Figure 5E,F).

## 4. Discussion

Despite favorable outcomes in patients after SAH, behavioral and cognitive dysfunction can persist, limiting daily activities [3,27]. Nevertheless, few studies have focused on the effect of stem cells on the recovery from these dysfunctions. Unlike SAH, the treatment of various neurological disorders with MSCs has been widely investigated [28,29,30]. Lee et al. [31] reported that patients with multiple-system atrophy treated with MSCs showed improvements in cerebellar dysfunction. Additionally, BMSC administration facilitated reductions in Aß and increased the expression of microRNA-146a in the hippocampus, reducing cognitive impairment [29,32]. Since neuroinflammation plays an important role in neurological disorders, we focused on the HMGB1–RAGE axis. Our findings showed that the expression of HMGB1 increased 6 h after the SAH induction. A single administration of BMSCs at 3 h reduced cognitive impairment while maintaining reductions in apoptosis and inflammatory cytokines within 24 h. However, these increased again after 72 h. Although the mild-SAH-model mice without BMSC administration showed some recovery after 72 h, the therapeutic effects were small. There was no difference on the NOR test between the saline- and BMSC-treated groups at 72 h. However, there was a difference at 72 h between multiple BMSC treatments and the saline treatment. Early brain injury (EBI) occurs immediately after SAH. Delayed cerebral ischemia (DCI) starts from 48 to 72 h after SAH ictus. It typically appears on day 4–10, unlike other forms of intracerebral hemorrhage. This characteristically causes inflammation and DCI, not immediately after SAH, but after some time [33]. Accordingly, additional BMSC treatment may be necessary to prevent secondary brain damage due to DCI, as well as to alleviate the inflammation caused by the initial brain damage in SAH to improve clinical symptoms, including cognitive impairment. Thus, administering BMSCs at different times could be more effective in restoring behavioral and cognitive dysfunction via persistent reductions in HMGB1–RAGE-mediated neuroinflammation. To confirm our hypothesis, we administered the BMSCs twice, at 3 h and 48 h after SAH induction, and compared the therapeutic effects to those of a single BMSC administration. Although the overall differences were not large, some improvements in inflammation and cognitive function were seen in the mild SAH. It is likely that the treatment effect would have been higher in cases of severe SAH. Therefore, it is thought that multiple BMSC treatments might be needed to prevent both EBI and DCI, rather than a single BMSC treatment, in order to improve neurological outcomes for SAH patients in actual clinical practice.

The optimal delivery method and therapeutic dose of BMSCs for SAH have not been determined. In this study, we selected an intravenous approach for BMSC transplantation, out of several delivery routes. Subarachnoid hemorrhage refers to bleeding that suddenly occurs in the subarachnoid space, which causes brain damage due to increased intracranial pressure. Thus, damage to the brain parenchyma is often not localized in patients with SAH compared to those with intracranial hemorrhage or traumatic brain injury. In addition, continuous cerebrospinal-fluid drainage (e.g., extraventricular drainage and lumbar drain) to relieve increased intracranial pressure is frequently performed after surgery. Overall, intravenous administration is more clinically useful and noninvasive than intraventricular or intraparenchymal approaches to BMSC transplantation following SAH. However, the trapping of BMSCs in the peripheral organs, particularly the lungs, is a concern after the administration of BMSCs [30]. Jung et al. [34] reported serial cases of pulmonary embolisms after multiple intravenous administrations of human-adipose-tissue-derived stem cells (AMSCs) for treating cervical herniated intervertebral discs. Although BMSCs with a molecular size of 7 μm exhibited a higher passage rate through the lungs than 18-μm AMSCs, it is necessary to design a delivery method that increases the number of BMSCs that reach the target brain region without being trapped in peripheral organs and that produces small-sized stem cells that pass through the BBB to increase the utility of BMSCs in patients with SAH in the future.

The optimal cell dosage of BMSCs for administration also remains undecided. However, it can be determined by the type of lesion and the purpose of the treatment. Zhang et al. [35] reported that a single administration of 3 to 4 × 10^5^ ADSCs improved neurological function by maintaining BBB stability after cerebral hemorrhage. Referring to the results of this study, we administered 3 × 10^5^ BMSCs to a mouse model of SAH. Nevertheless, varying doses should be tested to identify the therapeutic effects of stem cells in different species, as well as the optimal stem-cell type and delivery route. In addition, higher doses may be needed to identify stem cells’ effects in humans. An 80-year-old SAH patient experienced neurological improvement after receiving 1 × 10^7^ allogenic MSCs three days after ictus [36]. Therefore, it is necessary to study optimal doses according to the purpose and species, including human patients, in the future.

Administered MSCs were observed in damaged brains in a SAH model [11]. This finding suggests that MSCs migrated to an injured brain may be responsible for treatment efficacy and neuronal differentiation. The MSCs exhibited neuroplastic effects after injury by differentiating into glial cells, neurons, and endothelial cells after intravenous administration [11]. However, the main protective influence of BMSCs is thought to occur through their paracrine effects, particularly those of exosomes [7]. Xiong et al. [7] reported that the administration of exosomes derived from BMSCs improved neurological functions and reduced brain swelling via the anti-inflammatory effects of miRNA129-5p. In addition, exosomes secreted from BMSCs were taken up into astrocytes, which are involved in synapse formation [29]. Accordingly, investigations of the therapeutic effect of BMSC-derived exosomes in cognition improvement in SAH, particularly focusing on astrocytes, are required.

**Figure 6:**
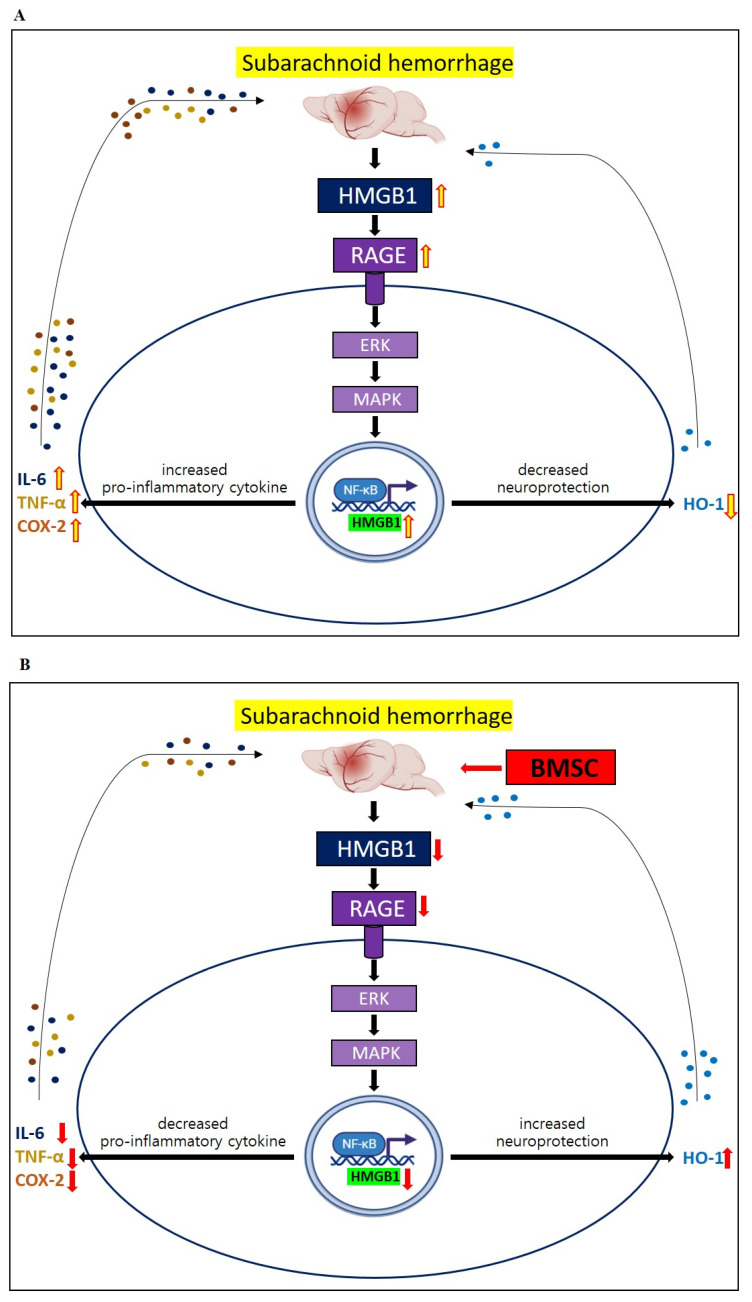
Schema showing therapeutic effects of saline administration (**A**) and BMSC administration (**B**) on SAH model mice in vivo. The administration of BMSCs may contribute to improvements in behavioral and cognitive dysfunction, mainly by relieving HMGB1–RAGE axis-mediated neuroinflammation.

This study has some limitations. First, we only enrolled mice with mild SAH and evaluated the therapeutic efficacy of the BMSCs based on neurological scores, not CT scans. When a severe-SAH model was generated, it was difficult to properly identify cognition changes due to the severe neurological damage. In addition, since CTs were not taken, varying amounts of SAH could have exerted similar effects on neurologic functioning. Accordingly, the results of this study were limited in that the cognitive-function-recovery effect was only seen in mice with mild SAH and a relatively good prognosis. Second, we focused on the EBI and analyzed the cognitive-function effects of BMSCs with a focus on reducing neuroinflammation via the HMGB-RAGE axis. Delayed cerebral ischemia, which mostly occurs between days 4 and 10 after ictus, can cause neurologic deterioration and aggravate neuroinflammation [33]. Eagles et al. [4] reported that DCI was a risk factor for behavioral and cognitive dysfunctions after SAH. Although DCI is more common in severe SAH, with a larger amount of initial bleeding than in mild SAH, further stem-cell studies are needed to reduce DCI in patients with mild SAH. Third, we did not compare the BMSC treatment to other treatments, but only found that the HMGB1–RAGE axis-mediated neuroinflammation was reduced by the administration of the BMSCs. Haruma et al. [37] reported that an anti-MGB1 antibody attenuated the activation of cerebrocortical microglia due to brain injury. Thus, a comparative analysis of the combined therapeutic efficacy of BMSCs and HMGB1 inhibition using small interfering RNA (siRNA) or monoclonal antibodies is needed in the future. Fourth, we did not evaluate how the BMSC administration affected the HMGB1/RAGE axis, and no significant effect on TLR4 was seen in the mice with mild SAH (Figure 6). In general, HMGB1 released from neuronal cells or glia cells activates RAGE and TLR4, resulting in neuroinflammation [14]. Zhang et al. [38] reported that siRNA-HMGB1 treatment decreased the expression of the RAGE protein, but not that of TLR4, in stress-induced microglial-mediated neuroinflammation. Accordingly, it is necessary to study the mechanism of how the administration of BMSCs contributes to decreasing neuroinflammation via the HMGB1–RAGE axis. Fifth, we harvested the brains using a standardized method for dissecting brain tissues while confirming the anatomical structure to ensure that the same brain regions were dissected from each mouse. Nevertheless, the brain-harvesting process can be imperfect, and these differences might have affected the results. Finally, we did not perform immunohistochemical staining to confirm the specific cellular localization of the increased mRNAs and proteins. While qRT-PCR and Western blotting are useful for quantifying gene- and protein-expression levels, they cannot provide information on the spatial distribution of these molecules within cells or tissues. Accordingly, future studies should investigate the use of immunohistochemical staining to localize the expression of mRNAs and proteins to determine the cellular and subcellular localizations of these molecules in cerebral arteries or brain tissues.

## 5. Conclusions

The administration of BMSCs reduced behavioral and cognitive dysfunction by ameliorating HMGB1–RAGE axis-mediated neuroinflammation after mild SAH in mice.

## Data Availability

Not applicable.

## Supplementary Materials

The following supporting information can be downloaded at: https://www.mdpi.com/article/10.3390/life13040881/s1, Figure S1: Experimental design of the study; Figure S2: Representative tracing images of NOR tests including training and memory tasks before SAH induction (A) and quantification of recognition in percentages (B,C); Table S1: Sequence of all primers used for qRT-PCR analysis; Figure S3: Raw data of Western blotting. References [39,40,41,42,43,44,45] are cited in Supplementary Materials.
