# Supplementary material for: Bone-Marrow-Derived Mesenchymal Stem Cells Attenuate Behavioral and Cognitive Dysfunction after Subarachnoid Hemorrhage via HMGB1–RAGE Axis Mediation
Source: Life (Basel). 2023 Mar 26;13(4):881. doi: 10.3390/life13040881 (PMC10145212; doi:10.3390/life13040881)

SUPPLEMENTAL METHODS

Rationales for investigating various markers used in this study are as follows: 1) IL-6 and TNF-α: These are pro-inflammatory cytokines that are known to be elevated after SAH induction. They are involved in the inflammatory response in the brain [1,2]. 2) COX2: COX2 is an enzyme that is involved in the production of prostaglandins, which can contribute to inflammation [3]. 3) HMGB1 and RAGE: HMGB1 is a protein that can activate the RAGE receptor, leading to inflammation and tissue damage [4]. 4) MyD88 and TLR4: These are proteins involved in the Toll-like receptor pathway, which plays a key role in innate immune response [5,6]. 5) NF-κB: NF-κB is a transcription factor that is activated in response to inflammation. It is involved in the expression of pro-inflammatory genes [7]. Overall, these markers were chosen based on their roles in inflammatory responses after SAH based on previous studies.

References

1. Ferenc Gallyas, Jr. Acute changes of pro-inflammatory markers and corticosterone in experimental subarachnoid haemorrhage: A prerequisite for severity assessment. PLoS One. 2019 (14): e0220467.

2. Sajjad Muhammad, Alexander Grote. Interleukin 6 and Aneurysmal Subarachnoid Hemorrhage. A Narrative Review. International Journal of Molecular Sciences. 2021 (22): 4133.

3. R Ayer. V Hadhav. T Sugawara. John H Zhang. The neuroprotective effects of cyclooxygenase-2 inhibition in a mouse model of aneurysmal subarachnoid hemorrhage. Acta Neurochir Suppl. 2011 (111): 145-149.

4. Yam Nath Paudel. Efthealia Angelopoulou. Christina Piperi. Lekhsan Othman. Mohd Farooq Shaikh. HMGB1-Mediated Neuroinflammatory Responses in Brain injuries: Potential Mechanisms and Therapeutic Opportunities. International Journal of Molecular Sciences. 2020 (21): 4609.

5. Lintao Wang. Guangping Geng. Tao Zhu. Wenwu Chen, et al. Progress in Research on TLR4-Mediated Inflammatory Response Mechanisms in Brain Injury after Subarachnoid Hemorrhage. Cells. 2022 (23): 3781.

6. Hammad Ahmed. Mahtab Ahmad Khan. Ulf Dietrich Kahlert. Mika Niemela, et al. Role of Adaptor Protein Myeloid Differentiation 88 (MyD88) in Post-Subarachnoid Hemorrhage: A Systematic Review. International Journal of Molecular Sciences. 2021 (22): 4185.

7. Anwen Shao. Haijian Wu. Yuan Hingm Sheng Yu. et al. Hydrogen-Rich Saline Attenuated Subarachnoid Hemorrhage-Induced Early Brain Injury in Rats by Suppressing Inflammatory Response: Possible Involvement of NF-kB Pathway and NLRP3 Inflammasome. Molecular Neurobiology. 2016 (53): 3462-3467.

SUPPLEMENTAL FIGURE LEGENDS

Supplemental Figure S1. Experimental design of the study.

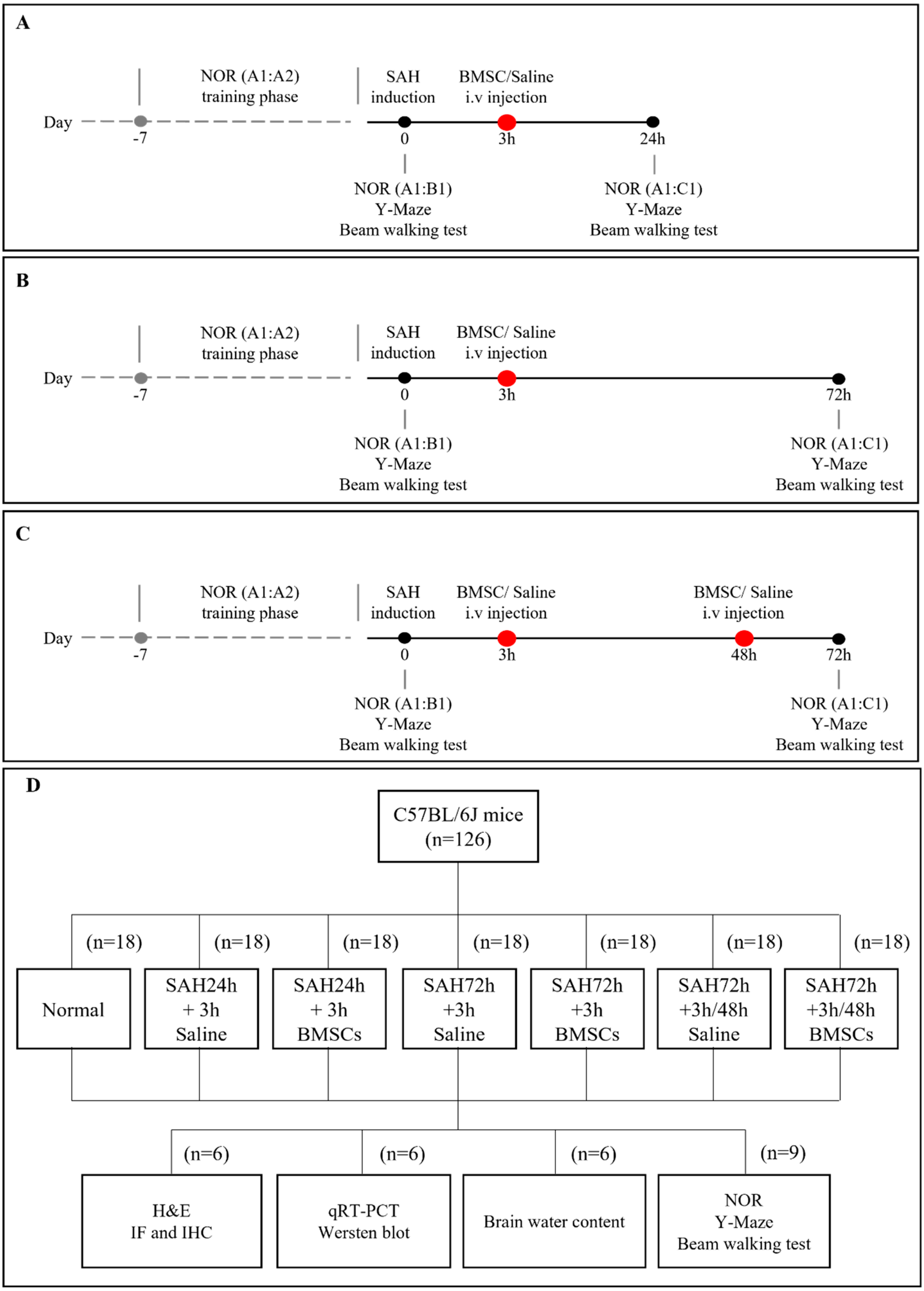

Supplemental Figure S2. Representative tracing images of NOR tests including training and memory tasks before SAH induction **(A)** and quantification of recognition in percentages **(B and C).**

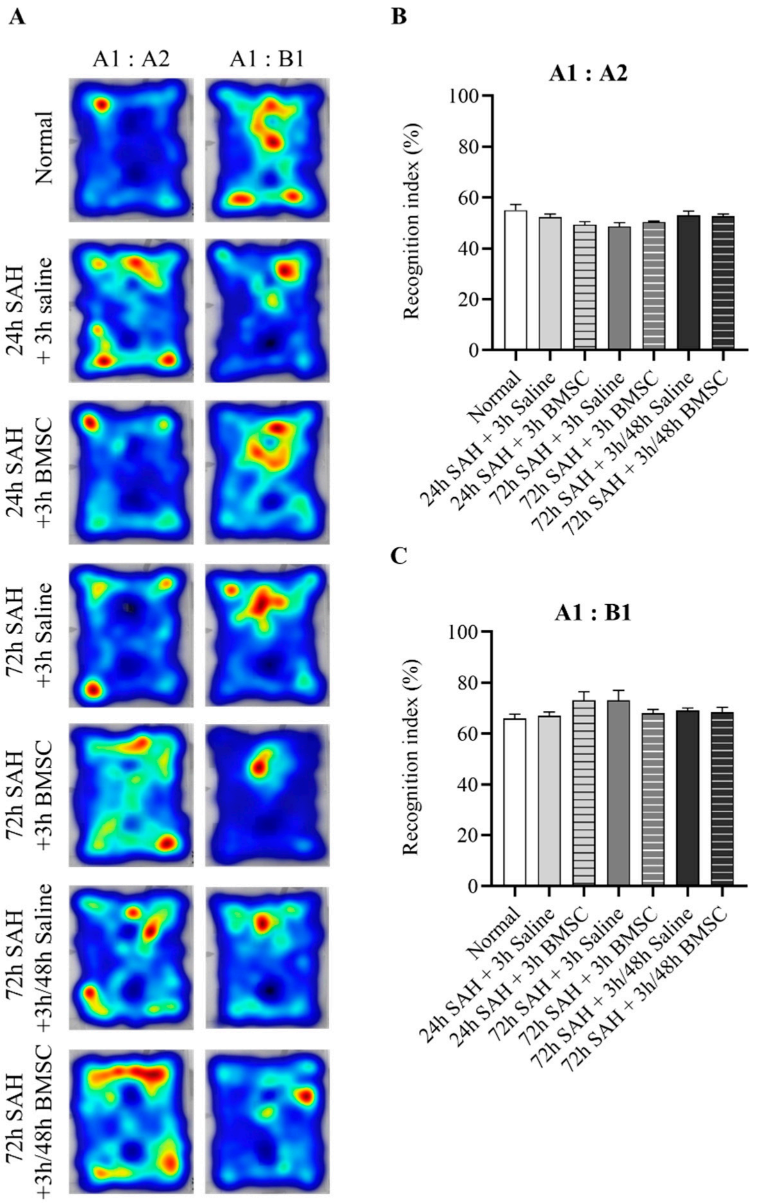

Supplemental Table S1. Sequence of all primers used for qRT-PCR analysis.

Genes		Sequences 5' -3'
IL-6	Forward	CCACGGCCTTCCCTACTT
	Reverse	TTGGGAGTGGTATCCTCTGTGA
TNF-α	Forward	GCTGTCCCTGCGCTTCA
	Reverse	CTCGTCCCCAATGACATCCT
COX-2	Forward	CGAGGCCACTGATACCTATTGC
	Reverse	GCTGGCCTGGTACTCAGTAGGTT
HMGB1	Forward	GCCTCGCGGAGGAAAATC
	Reverse	AAGTTTGCACAAAGAATGCATATG
RAGE	Forward	TCAACATCAGGGTCACAGAAAC
	Reverse	CAATGAGCAGAGCGGCTA
TLR4	Forward	GCCTTTCAGGGAATTAAGCTCC
	Reverse	AGATCAACCGATGGACGTGTAA
MyD88	Forward	TCATGTTCTCCATACCCTTGGT
	Reverse	AAACTGCGAGTGGGGTCAG
GAPDH	Forward	TTGATGGCAACAATCTTCAC
	Reverse	CGTCCCGTAGACAAAATGGT

Supplemental Figure S3. Raw data of western blotting.

A. IL-6 for Fig 2G

IL-6 (21 kDa) β-actin (43 kDa)

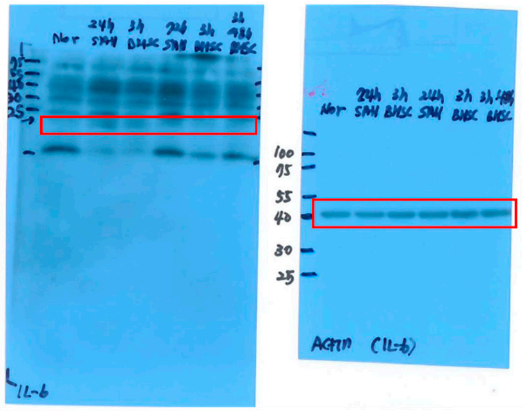

B. TNF-α for Fig 2H

TNF-α (26 and 17 kDa) β -actin (43 kDa)

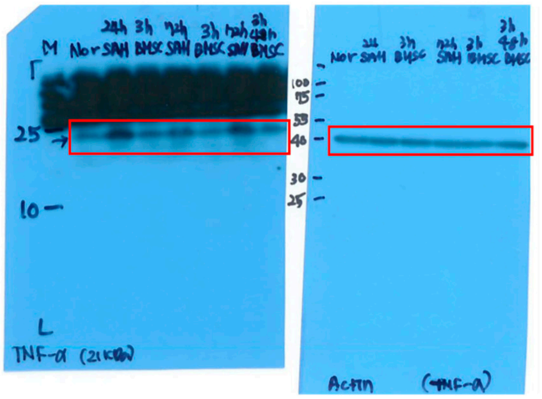

C. COX-2 for Fig 2I

COX-2 (69 kDa) β-actin (43 kDa)

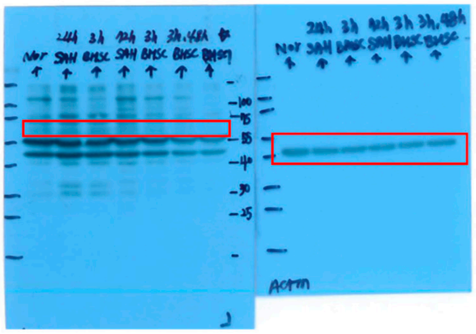

D. HMGB1 for Fig 3E

COX-2 (29 kDa) β-actin (43 kDa)

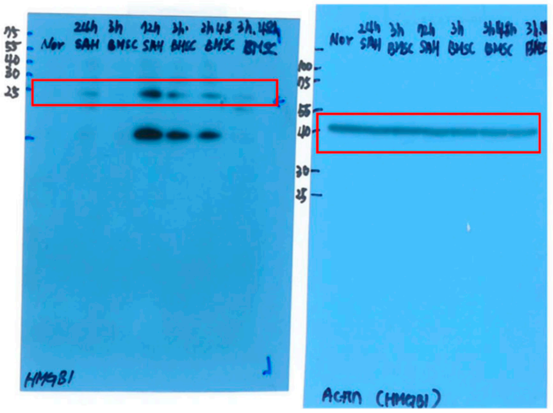

E. Phospho NFkB p65 and NFkB p65 for Fig 3F

Phospho NF-kB p65 (65 kDa) NF-kB p65 (65 kDa) β-actin (43 kDa)

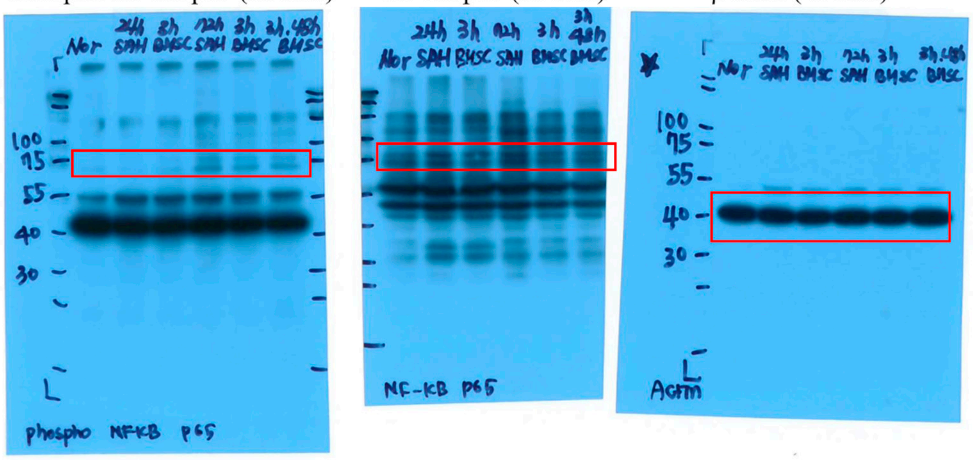

Supplement: Supplementary file 1 [file life-13-00881-s001.zip › life-2290732-supplementary.pdf]
